# Functional and free-water imaging in rapid eye movement behaviour disorder and Parkinson’s disease

**DOI:** 10.1093/braincomms/fcae344

**Published:** 2024-10-10

**Authors:** Emily R Tobin, David J Arpin, Marissa B Schauder, Mara L Higgonbottham, Robin Chen, XiangYang Lou, Richard B Berry, Evangelos A Christou, Michael S Jaffee, David E Vaillancourt

**Affiliations:** Department of Applied Physiology and Kinesiology, University of Florida, Gainesville, FL 32611, USA; Department of Applied Physiology and Kinesiology, University of Florida, Gainesville, FL 32611, USA; Department of Applied Physiology and Kinesiology, University of Florida, Gainesville, FL 32611, USA; Department of Applied Physiology and Kinesiology, University of Florida, Gainesville, FL 32611, USA; Department of Biomedical Engineering, University of Florida, Gainesville, FL 32603, USA; Department of Biostatistics, University of Florida, Gainesville, FL 32603, USA; Department of Medicine, Division of Pulmonary, Critical Care and Sleep, University of Florida, Gainesville, FL 32610, USA; Department of Applied Physiology and Kinesiology, University of Florida, Gainesville, FL 32611, USA; Department of Biomedical Engineering, University of Florida, Gainesville, FL 32603, USA; Fixel Institute for Neurological Disease, University of Florida, Gainesville, FL 32608, USA; Department of Applied Physiology and Kinesiology, University of Florida, Gainesville, FL 32611, USA; Department of Biomedical Engineering, University of Florida, Gainesville, FL 32603, USA; Fixel Institute for Neurological Disease, University of Florida, Gainesville, FL 32608, USA

**Keywords:** REM sleep behaviour disorder, Parkinson’s disease, fMRI, free-water, Purdue Pegboard Test

## Abstract

It is established that one of the best predictors of a future diagnosis of Parkinson’s disease is a current diagnosis of rapid eye movement behaviour disorder (RBD). In such patients, this provides a unique opportunity to study brain physiology and behavioural motor features of RBD that may precede early-stage Parkinson’s disease. Based on prior work in early-stage Parkinson’s disease, we aim to determine if the function of corticostriatal and cerebellar regions are impaired in RBD using task-based functional MRI and if structural changes can be detected within the caudate, putamen and substantia nigra in RBD using free-water imaging. To assess motor function, we measured performance on the Purdue Pegboard Test, which is affected in patients with RBD and Parkinson’s disease. A cohort of 24 RBD, 39 early-stage Parkinson’s disease and 25 controls were investigated. All participants were imaged at 3 Telsa. Individuals performed a unimanual grip force task during functional imaging. Participants also completed scales to assess cognition, sleep and motor symptoms. We found decreased functional activity in both RBD and Parkinson’s disease within the motor cortex, caudate, putamen and thalamus compared with controls. There was elevated free-water-corrected fractional anisotropy in the putamen in RBD and Parkinson’s disease and elevated free-water in the putamen and posterior substantia nigra in Parkinson’s disease compared with controls. Participants with RBD and Parkinson’s disease performed significantly worse on all tasks of the Purdue Pegboard Test compared with controls. The both hands task of the Purdue Pegboard Test was most sensitive in distinguishing between groups. A subgroup analysis of early-stage RBD (<2 years diagnosis) confirmed similar findings as those in the larger RBD group. These findings provide new evidence that the putamen is affected in early-stage RBD using both functional and free-water imaging. We also found evidence that the striatum, thalamus and motor cortex have reduced functional activity in early-stage RBD and Parkinson’s disease. While the substantia nigra shows elevated free-water in Parkinson’s disease, we did not observe this effect in early-stage RBD. These findings point to the corticostriatal and thalamocortical circuits being impaired in RBD patients.

## Teaser

Tobin *et al*. studied early-stage rapid eye movement behaviour disorder (RBD) and Parkinson’s disease using task-based functional magnetic resonance imaging and free-water imaging. New evidence is provided that the putamen is affected in early-stage RBD for both functional and free-water imaging, and the corticostriatal and thalamocortical circuits are functionally impaired in RBD.

## Graphical Abstract

**Graphical Abstract:**
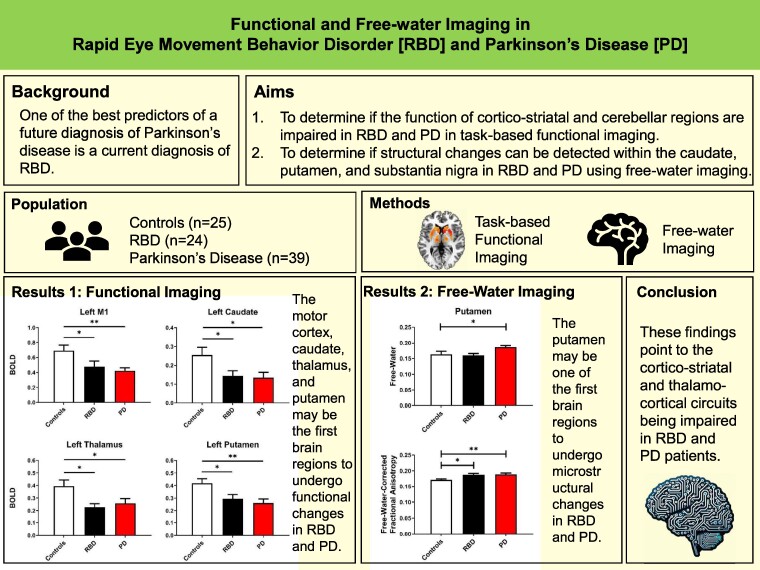

## Introduction

Clinical diagnosis of Parkinson’s disease occurs following the appearance of motor symptoms,^1^ by which point 50–70% of the nigral dopaminergic neurons have already degenerated.^2-4^ The motor features of Parkinson’s disease include bradykinesia, tremor, rigidity and postural impairments. Several prodromal features of Parkinson’s disease may occur prior to the appearance of motor symptoms, including constipation, olfactory loss, sexual dysfunction and daytime sleepiness.^5-9^ The most predictive prodromal feature though is a diagnosis of rapid eye movement (REM) behaviour disorder (RBD).^5,10,11^ While Parkinson’s disease patients can have RBD, and thus would have motor deficits, far less is known about the possible changes in patients with RBD that have not converted to Parkinson’s disease. In a prospective large multisite study, Postuma *et al*.^12^ found that the conversion rate from RBD to an overt neurodegenerative syndrome was 73.5% after a 12-year follow-up. Similar to other neurodegenerative syndromes, motor abnormalities often occur in RBD, including limb bradykinesia, reduced arm swing and tremor.^6,13^ A key unanswered question in the understanding of RBD is how brain function and structure are altered before conversion to an overt neurodegenerative syndrome.^14,15^ Addressing this question will provide novel information about the pathophysiology and neurodegenerative changes associated with RBD. Here, we explore corticostriatal, nigrostriatal, thalamic and cerebellar changes in RBD using task-based functional MRI (fMRI) and structural free-water imaging.

Task-based fMRI has yielded robust findings indicating that individuals with early-stage Parkinson’s disease have reduced blood-oxygen-level-dependent (BOLD) activity in the cortex, caudate, putamen, thalamus and cerebellum compared with controls.^16-25^ In addition, prior work found that the putamen and primary motor cortex (M1) BOLD activity decreased longitudinally over 1-year in early-stage Parkinson’s disease.^21^ Dopamine transporter (DAT) imaging and other PET radiotracers have also shown that the presynaptic dopaminergic nerve terminals are affected in Parkinson’s disease^26-29^ and RBD.^30-33^ Utilizing DAT imaging, Zoetmulder *et al*.^34^ found that dopamine uptake in the putamen was highest in controls, followed by RBD, and lowest in Parkinson’s disease. In addition, prior work has found longitudinal reduction in striatal DAT binding over 1, 2 and 4 years of disease progression in early-stage Parkinson’s disease^35^ and over 3 years of disease progression in RBD.^36^

Free-water imaging is a diffusion-weighted imaging technique that utilizes a two-compartment model to separate the diffusion properties of water in brain tissue from those of water in the extracellular space (free-water).^37^ This procedure allows the calculation of the fractional volume of the extracellular compartment (free-water) as well as the fractional anisotropy of the free-water-corrected intracellular (tissue) compartment (FA_T_) within a voxel. Free-water imaging has yielded robust findings indicating elevated free-water in the posterior substantia nigra (pSN) in early-stage Parkinson’s disease,^38-41^ which continues to increase longitudinally.^42,43^ In addition, prior work found that individuals with RBD have intermediate levels of pSN free-water between controls and Parkinson’s disease, suggesting that the pSN is affected well before a Parkinson’s disease diagnosis.^44-46^ Elevated putamen free-water values have also been found in atypical parkinsonism^38,47^ and RBD^46^ compared with controls. Increased FA_T_ values have been shown in the putamen and caudate in atypical parkinsonism^38^ and in the pSN in Parkinson’s disease^40,42^ compared with controls. Elevated levels of free-water and FA_T_ are linked to neuroinflammation and/or an expanded extracellular partial volume resulting from a reduction in tissue compartment volume.^47-50^ The effectiveness of free-water and other neuroimaging techniques as a potential biomarker for preclinical/prodromal Parkinson’s disease, early-stage Parkinson’s disease and moderate to late-stage Parkinson’s disease has been summarized in a prior review article.^31^

It is currently unknown whether functional and free-water changes in the basal ganglia occur simultaneously or in a sequential order. If free-water changes in the pSN are affected without functional or free-water changes in the putamen, this would suggest that pSN free-water may represent one of the earliest changes in RBD. In contrast, it is possible that functional and free-water changes occur in the putamen prior to the degeneration in the pSN. Determining which neuroanatomical region changes first could lead to the development of better biomarkers, which could result in earlier diagnoses and more effective treatment options for both RBD and Parkinson’s disease.

The Purdue Pegboard Test (PPT) is an objective, easy-to-administer, cost-effective and reliable way to classify upper extremity motor deficits in Parkinson’s disease and has been shown to track disease progression.^51-54^ Recent studies found that individuals with probable RBD or idiopathic RBD had intermediate PPT composite scores between controls and Parkinson’s disease.^55-57^ Since composite scores have been used to assess PPT performance in RBD, it is unclear if any single PPT task is able to differentiate between groups. Wilkes *et al*.^51^ found that neuroimaging measures were associated with the both hands PPT score in Parkinson’s disease and atypical parkinsonism; however, it is unknown if neuroimaging measures are associated with PPT scores in RBD.

Here, we investigate whether the motor cortex, pSN, putamen, caudate, thalamus and cerebellum differ using task-based functional imaging and free-water imaging in RBD compared with a healthy control group. Furthermore, we contrast the changes observed in RBD with those observed in early-stage Parkinson’s disease. In addition, we examine if functional and free-water imaging is associated with PPT performance and Part 3 of the Movement Disorders Society-Unified Parkinson’s Disease Rating Scale (MDS-UPDRS-III) in controls, RBD and Parkinson’s disease participants.

## Methods

### Participants

The study included 25 control participants, 24 participants with RBD and 39 participants with Parkinson’s disease. All participants were between the ages of 40 and 75. One Parkinson’s disease participant was excluded from the free-water imaging analysis as the free-water scan was not collected for this individual. When selecting participants, candidates were excluded for active seizures, stroke, brain tumour or implanted pacemaker or neurostimulator. When selecting controls, candidates were also excluded if they had a family history of Parkinson’s disease or had a previous traumatic brain injury. Parkinson’s disease participants were referred from and diagnosed by movement disorder specialists at the Fixel Institute for Neurological Disease at the University of Florida. Parkinson’s disease participants were ≤2 Hoehn and Yahr stage when on medication. RBD participants were referred by the sleep medicine clinic associated with UF Health and included patients who met the criteria for RBD as defined in the ICSD-3-TR and who completed a polysomnography sleep study.^58^ The patients enrolled in the RBD cohort did not meet the criteria for any other sleep disorder, including sleep-disordered breathing disorders or disorders of hypersomnolence such as narcolepsy. Patients with pathology on a brain MRI that could account for symptoms were excluded from the study. While we did not stratify patients who may have been on antidepressant medications, it should be noted that antidepressants have been associated more with REM sleep without atonia than with the symptomatic criteria of RBD.^59^ Our cohort included patients who met both the clinical and polysomnography criteria for RBD. Participants who were on antiparkinsonian (dopaminergic) medication performed all testing after overnight withdrawal of >12 h. Controls were recruited by word of mouth and from the surrounding community and were age and sex matched to Parkinson’s disease participants. All procedures were approved by the Institutional Review Board, and written informed consent was obtained from all participants in accordance with the Declaration of Helsinki.

### Clinical assessment

Cognitive function was assessed by using the Montreal Cognitive Assessment (MOCA).^60^ The REM sleep behaviour disorder questionnaire (RBDSQ) was used to further assess and classify sleep behaviour.^61^ Participants were administered the MDS-UPDRS-III to classify motor function.^62^ The PPT was used to assess motor control, which includes four tasks (dominant hand, non-dominant hand, both hands simultaneously and assembly).^51,63^

### FMRI force task

**Figure 1:**
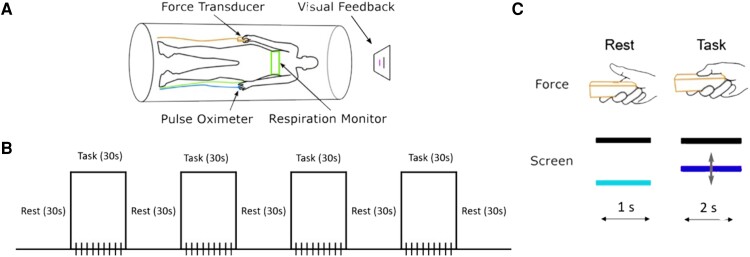
**fMRI task.** (**A**) Participant set-up inside the scanner. All participants were positioned lying on their back inside the scanner with the force sensor placed in their right hand, a pulse oximeter positioned on the index finger of the left hand and a respiratory belt monitor placed across the subject’s chest. (**B**) fMRI block task design. One scan consists of a 30-s rest block followed by a 30-s task block. This sequence will be repeated four times, and then an additional 30-s rest block will be added on at the end. (**C**) Force task. Position and grip of the right-hand during rest condition and force production part of the task. One bar is a white stationary/target bar, which marks 15% of the individuals MVC and does not move during the task. The other bar changes from light blue to navy blue and represents the force bar. When the bar is light blue, this indicates that the participant should relax their hand and stop producing force. During the force production part of the task, when the bar turned to navy blue, participants are instructed to press on the sensor, moving the bar vertical to align and hold it on the top of the white target bar. The navy blue force bar also instructs participants to generate and maintain 15% MVC force for 2 s, and the light blue force bar instructs the participants to release force for 1 s.

A unilateral precision grip force task was used to assess cerebral and cerebellar activity during functional imaging. All participants were positioned lying on their back inside the scanner with the force sensor placed in their right hand, a pulse oximeter positioned on the index finger of the left hand and a respiratory belt monitor placed across the subject’s chest (Fig. 1A). Visual feedback was displayed on an LCD monitor that participants could see through a mirror mounted on the head coil. The task-based fMRI study was a block design, which consisted of a 30-s rest block followed by a 30-s task block. This sequence was repeated four times and an additional 30-s rest block was added, for a total scan time of 4.5 min (Fig. 1B). A total of three task-based fMRI scans were collected for each participant. The visual feedback consisted of two bars displayed on a black background. One bar was a white stationary/target bar, which marked 15% of the individual's maximum voluntary contraction (MVC). The other bar changed from light blue to navy blue and moved vertically based on the amount of force produced. When the bar was light blue, this indicated that the participant should relax their hand and stop producing force. When the bar turned navy blue, this indicated that the participant should press on the force sensor, moving the bar vertically to align and hold it on top of the white target bar. The bar was navy blue for 2 s and light bluefor 1 s and repeated this alternating sequence 10 times during one 30-s task block (Fig. 1C).

Grip force control was measured using a custom-designed MRI-compatible fibre optic transducer with a resolution of 0.025 N (Neuroimaging Solutions, Gainesville, FL, USA). Force data were sampled at 125 Hz by a sm130 Fiber Optic Interrogator (Micron Optics, Atlanta, GA, USA) and recorded using LabVIEW (National Instruments, Austin, TX, USA) (Neuroimaging Solutions) software. The force data were filtered using Neuroimaging Solutions software, and the % MVC was calculated by finding the peak force and the five samples around the peak force and averaging these values across three trials. The fMRI task focuses on contraction and relaxation of the hand musculature, which is well established to drive the motor cortex, basal ganglia and cerebellum, and reveals established corticostriatal deficits in Parkinson’s disease.^19,20,22,23,25^ The outcome measure of the task-based fMRI was the difference in BOLD signal during force generation and rest.

### MRI acquisition

Data were collected on a 3 T Philips system (Best, the Netherlands) with a 32-channel SENSE head coil. The protocol included an anatomical 3D T1-weighted sequence (repetition time = 9.8 ms, echo time = 4.5 ms, flip angle = 8^0^, field of view = 256 mm^2^ and voxel size = 0.8 mm isotropic). The free-water-weighted scan consisted of the following parameters: repetition time = 6400 ms, echo time = 86 ms, flip angle = 70^0^, field of view = 256 × 256 mm, resolution = 2 mm isotropic, 64 diffusion gradient directions, two *b*-values = 0 (5 average) and 1000 s/mm^2^ and 72 axial slices. The fMRI protocol included a T2*-weighted, single-shot, echoplanar pulse sequence repetition time = 3000 ms, echo time = 30 ms, flip angle = 80^0^, field of view = 240 mm^2^ and voxel size = 3 mm (isotropic).

### MRI analysis

Functional imaging processing steps were based on previous fMRI studies of grip force in parkinsonian disorders.^19-23,25,64,65^ All participants used their right hand to produce force. Task-based BOLD activity was quantified in the following prespecified regions of interest (ROIs): left M1, left supplementary motor area (SMA), left caudate, left anterior dorsal putamen, left anterior ventral putamen, left posterior dorsal putamen, left posterior ventral putamen, left thalamus, right SMA, right caudate, right anterior dorsal putamen, right anterior ventral putamen, right posterior dorsal putamen, right posterior ventral putamen, right thalamus and right superior cerebellum (lobule V). These ROIs were selected based on previous findings of decreased BOLD activity in Parkinson’s disease and atypical parkinsonism compared with controls.^19-24^ The SMA ROI was taken from the Human Motor Area Template,^66^ and the caudate and putamen ROIs were from the Basal Ganglia Human Area Template.^67^ The putamen was then split into four segments: anterior dorsal, anterior ventral, posterior dorsal and posterior ventral. To obtain the left anterior/posterior split, the putamen was separated at Montreal Neurological Institute space coordinate *y* = 1. To obtain the left dorsal/ventral split, the putamen was separated at Montreal Neurological Institute space coordinate *z* = 1. We used 3dLRflip in AFNI to obtain the same four segments on the right side of the brain. The thalamus ROI was created by combining all the subdivisions of the thalamus in the automatic anatomical labelling atlas 3.^68^ The left M1 and right superior cerebellum ROIs were obtained from previously published studies.^19,21^

Free-water-weighted images were preprocessed using FSL (fsl.fmrib. ox.ac.uk)^69-71^ for eddy current correction, head motion correction (using an affine registration) and brain extraction.^69^ The free-water analysis was performed using software from Neuropacs Corp and outlined in prior work.^37,39,41,42,72,73^ We calculated free-water maps for each participant before applying non-linear transformations to warp the free-water maps to a template in standard Montreal Neurological Institute space. Free-water and FA_T_ means (± SD) were calculated for the following prespecified ROIs: pSN, putamen and caudate. These ROIs were selected based on previous work showing increased free-water in Parkinson’s disease compared with controls within these regions.^38,39,43,73^ The pSN, putamen and caudate ROIs used here were validated against hand-drawn regions in prior work.^74^ The ROIs were averaged across left and right hemispheres.

### Statistical analysis

Descriptive statistics for demographic characteristics and clinical outcomes were calculated as the mean ± standard deviation for continuous variables and as counts for categorical variables. To evaluate differences between controls, RBD and Parkinson’s disease in age, MVC, MOCA, RBDSQ and MDS-UPDRS-III, we performed one-way ANOVAs. We performed a *t*-test to determine differences in the average length of diagnosis between RBD and Parkinson’s disease for this cohort. Fisher’s exact test was used to detect differences in categorical demographic variables (handedness and sex). For the BOLD analysis, we performed a three-factor repeated measures [group (3) × scan (3)] multivariate analysis of covariance (MANCOVA), which included each ROI (left M1, left SMA, left caudate, left anterior dorsal putamen, left anterior ventral putamen, left posterior dorsal putamen, left posterior ventral putamen, left thalamus, right SMA, right caudate, right anterior dorsal putamen, right anterior ventral putamen, right posterior dorsal putamen, right posterior ventral putamen, right thalamus and right superior cerebellum). Group (controls, RBD and Parkinson’s disease) was a between-subjects factor, and scan (Scan 1, Scan 2 and Scan 3) was a within-subjects factor. Age, sex and handedness were included as covariates. To evaluate group differences in free-water, FA_T_ and PPT, we performed MANCOVAs with age and sex as covariates. We also calculated the effect size for the different PPT tasks to determine which task was the most sensitive to changes between the groups. Pairwise *post hoc* comparisons for BOLD imaging, free-water imaging and the PPT were corrected for multiple comparisons using the Benjamini and Hochberg false discovery rate (FDR) and considered significant when *P*_FDR_ < 0.05.^75^

To observe the relationship between scores on the PPT and functional (BOLD) imaging and scores on the MDS-UPDRS-III and functional imaging, we performed backward linear regressions using the BOLD ROIs that significantly differed between Parkinson’s disease and control (PD-CON-BOLD ROIs) for each group (controls, RBD and Parkinson’s disease). A total of six regressions were run for BOLD imaging. For the regressions using PPT score, we selected to use only the scores from the both hands task as the dependent variable since the BOLD imaging data were derived from ROIs in both hemispheres of the brain and because we found that the both hands task was the most sensitive PPT task in this study. To correlate PPT both hands scores with BOLD imaging, we entered significant PD-CON-BOLD ROIs into the initial model (left M1, left caudate, left anterior dorsal putamen, left anterior ventral putamen, left posterior dorsal putamen, left thalamus, right caudate, right anterior dorsal putamen, righ anterior ventral putamen, right posteriror dorsal putamen, right thalamus and right superior cerebellum) as well as age and sex and performed three separate regressions, one for each of the groups (controls, RBD and Parkinson’s disease). In addition, to correlate MDS-UPDRS-III scores with BOLD imaging, we utilized the same 14 variables listed above and performed three separate regressions. At each step, the least significant factor was removed from the regression model until only those with *P* < 0.05 remained. The model that resulted in the lowest Akaike information criterion was selected as the best-fit model.^76^ Multicollinearity in the resulting models was quantified using the variance inflation factor (VIF), and variables with VIF > 10 were removed.

To observe the relationship between the scores on both hands task of the PPT and free-water imaging (free-water and FA_T_) and scores on the MDS-UPDRS-III and free-water imaging, we performed backward linear regression using the significant Parkinson’s disease versus control free-water ROIs (PD-CON-FW-FA_T_ ROIs) for each group (controls, RBD and Parkinson’s disease). In total, six regressions were run for free-water imaging. Specifically, to correlate PPT both hands score with free-water imaging, we entered the significant PD-CON-FW-FA_T_ ROIs into the model (putamen free-water, SN free-water and putamen FA_T_), as well as age and sex and performed three separate regressions, one for each of the groups (controls, RBD and Parkinson’s disease). Similarly, to correlate MDS-UPDRS-III score with free-water imaging, we utilized the same five variables listed above and performed three separate regressions. The best model was reported based on the criteria previously mentioned. Multicollinearity was assessed in the final model, and variables were removed if VIF > 10.

As a secondary analysis, to further understand changes in brain physiology and behavioural motor features in early-stage RBD, we used a subset of 16 participants with RBD and 25 controls. Participants with RBD were included if they were diagnosed within 2 years of testing. The same statistical analysis was used for the demographic, functional imaging, free-water imaging and the PPT data as was performed in the primary analysis. Additionally, we explored whether the most affected side in Parkinson’s disease participants impacted the performance of the dominant and non-dominant hand during the PPT. We split the Parkinson’s disease participants (*n* = 39) into two groups based on MDS-UPDRS-III subsections. In Group 1, the dominant hand matched the most affected side (PD-Dom-Dom), while in Group 2, the dominant hand did not match the most affected side (PD-Dom-NonDom). The groups for this secondary analysis included the 25 controls, 24 RBD, 21 PD-Dom-Dom and 18 PD-Dom-NonDom. The same statistical analysis was run on the demographic data and the PPT as was used in the primary analysis.

Statistical analysis was performed in SPSS version 29 for the repeated measures MANCOVA, MANCOVAs and backward linear regressions. Statistical analysis was performed in R version 4.3.0 (2023-04-21) and RStudio version 2023.12.1 + 402 (released 2024-01-29) for demographic data and pairwise *post hoc* analysis. All test statistics were performed as two tailed, where applicable. One-way ANOVA’s used Type 3 sum of squares.

## Results

### Demographic data

A summary of demographic data from our cohort can be found in Table 1. There were no significant group differences in age (*P* = 0.424), sex (*P* = 0.091), MVC (*P* =0.845) or handedness (*P* = 0.060). Disease duration was not different between RBD and Parkinson’s disease [*t*(60) = −1.514, *P* = 0.135]. We found significant group differences in RBDSQ score, MOCA score and MDS-UPDRS-III score (*P*’s < 0.05). In *post hoc* analysis, we found that controls had significantly lower RBDSQ scores compared with RBD (*P*_FDR_ < 0.001) and Parkinson’s disease (*P*_FDR_ = 0.004). RBD had significantly higher RBDSQ scores compared with Parkinson’s disease (*P*_FDR_ < 0.001). Controls had significantly higher MOCA scores compared with RBD and Parkinson’s disease (*P*_FDR_’s = 0.027). Parkinson’s disease had significantly higher MDS-UPDRS-III scores compared with controls and RBD (*P*_FDR_’s < 0.001). RBD had significantly higher MDS-UPDRS-III scores compared with controls (*P*_FDR_ = 0.033). All other pairwise *post hoc* comparisons did not significantly differ (*P*_FDR_ > 0.05).

**Table 1:** Participant demographic information

Demographics	Controls (*n* = 25)	RBD (*n* = 24)	Parkinson’s Disease (*n* = 39)	*P*-value
Age	62.56 ± 7.880	60.29 ± 8.595	62.95 ± 7.780	0.424
Sex	11M/14F	18M/6F	23M/16F	0.091
Handedness	20R/5L	24R/0L	34R/5L	0.225
Disease duration (months)	-	21.26 ± 27.83	29.87 ± 17.03	0.135
MVC	79.90 ± 23.01	84.26 ± 28.13	81.07 ± 29.28	0.845
RBDSQ	1.400 ± 1.607	7.460 ± 2.502	3.050 ± 2.224	<0.001***
MOCA	27.00 ± 2.179	25.25 ± 3.040	25.36 ± 2.622	0.023*
MDS-UPDRS-III	4.320 ± 3.301	8.210 ± 7.779	16.62 ± 6.683	<0.001***

Participant’s sex, handedness, disease duration, age and mean scores (SD) from the participant’s MVC, RBDSQ, MOCA and MDS-UPDRS-III. One-way ANOVA was performed for continuous data and Fisher’s exact test was performed for categorical data to determine group differences. Significance indicated by *P < 0.05 and ***P < 0.001.

MDS-UPDRS-III, Movement Disorder’s Society-Unified Parkinson’s Rating Disease Scale, Part III; MOCA, Montreal Cognitive Assessment; MVC, maximum voluntary contraction; RBD, rapid eye movement behaviour disorder; RBDSQ, rapid eye movement sleep questionnaire; SD, standard deviation; SMA, supplementary motor area.

### Functional imaging

**Figure 2:**
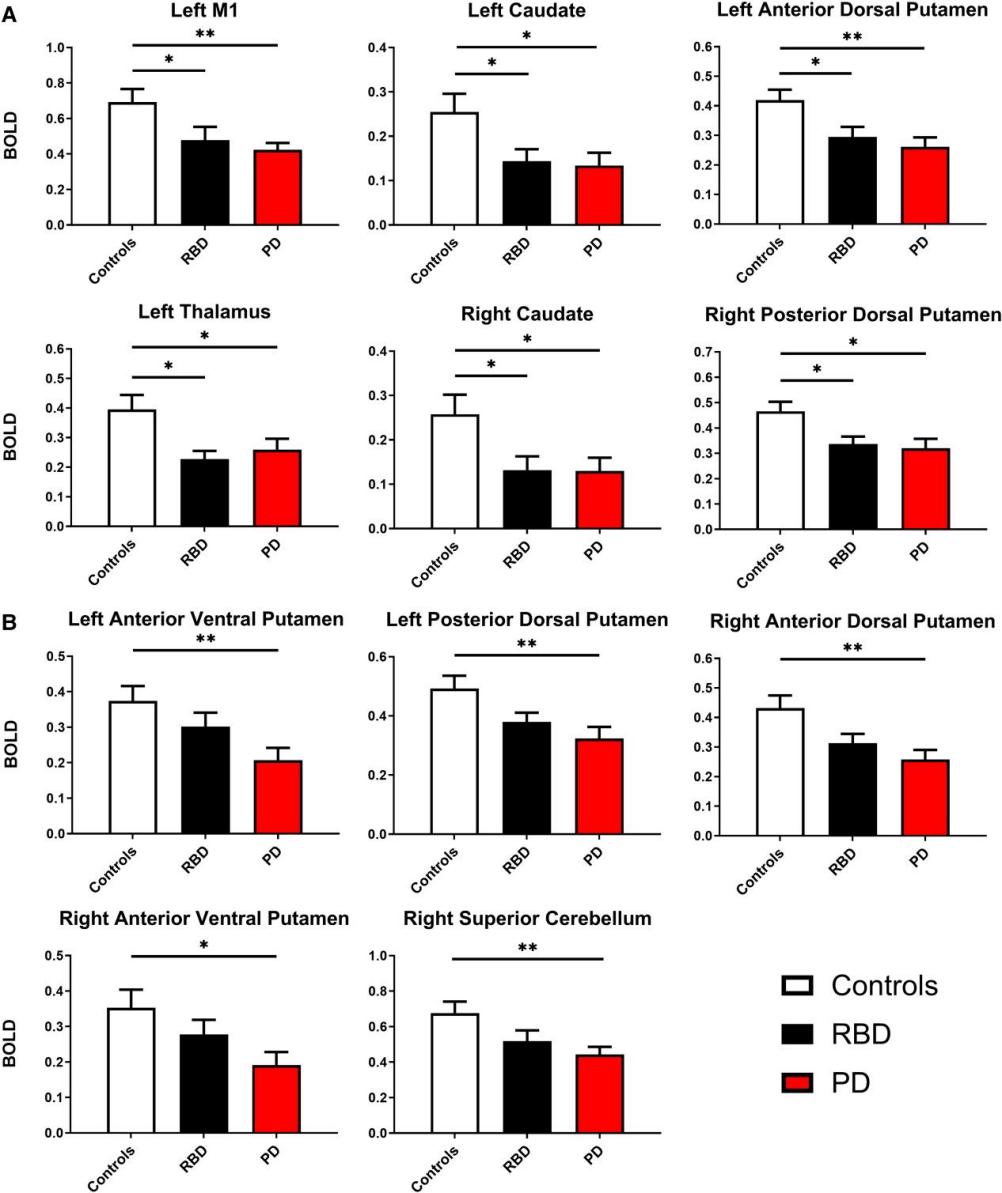
**BOLD imaging *post hoc*.** (**A**) Group means (± 1 SEM) for controls versus RBD and controls versus Parkinson’s disease for significant BOLD ROIs. (**B**) Group means (± 1 SEM) for only controls versus Parkinson’s disease for significant BOLD ROIs. Significance indicated by *P_FDR_ < 0.05 and **P_FDR_ < 0.01. Note: Repeated measures MANCOVA was used covarying for age, sex and handedness. Controls (*n* = 25). RBD (*n* = 24). Parkinson’s disease (*n* = 39). FDR, false discovery rate; MANCOVA, multivariate analysis of covariance; M1, primary motor cortex; PD, Parkinson’s disease; RBD, rapid eye movement behaviour disorder; SEM, standard error of the mean.

To evaluate significant differences in the BOLD response, a three-factor [group (3) × scan (3)] repeated measures MANCOVA was used covarying for age, sex and handedness. The main effect of scan [*F*(32,51) = 0.780, *P* = 0.770, Wilks’ Λ = 0.671] and the interaction effects between scan and group [*F*(64,102) = 0.998, *P* = 0.497, Wilks’ Λ = 0.378] were not significant. There was a statistically significant difference between groups for the overall MANCOVA model [*F*(32,134) = 1.704, *P* = 0.017, Wilks’ Λ = 0.502]. The following ROIs showed a significant main effect of group: left M1, left caudate, left anterior dorsal putamen, left anterior ventral putamen, left posterior dorsal putamen, left thalamus, right caudate, right anterior dorsal putamen, right anterior ventral putamen, right posterior dorsal putamen, right thalamus and right superior cerebellum (*P*’s < 0.05) (Table 2). In *post hoc* analysis, we found that controls have significantly increased BOLD signal compared with Parkinson’s disease (*P*_FDR_’s < 0.05) and RBD (*P*_FDR_’s < 0.05) in left M1, left caudate, left anterior dorsal putamen, left thalamus, right caudate and right posterior dorsal putamen (Fig. 2A). In addition, we found that controls have increased BOLD signal compared with Parkinson’s disease (*P*_FDR_’s < 0.05) in the left anterior ventral putamen, left posterior dorsal putamen, right anterior dorsal putamen, right anterior ventral putamen and right superior cerebellum (Fig. 2B).

**Table 2:** Functional imaging data

Brain region of interest	Controls (*n* = 25)	RBD (*n* = 24)	Parkinson’s disease (*n* = 39)	Diagnosis (*P*-value)	Scan (*P*-value)	Interaction scan * diagnosis (*P*-value)
Left M1	0.693 ± 0.372	0.478 ± 0.366	0.424 ± 0.237	0.002**^,a,b^	0.100	0.259
Left SMA	0.549 ± 0.303	0.554 ± 0.350	0.491 ± 0.288	0.558	0.228	0.174
Left Caudate	0.255 ± 0.205	0.144 ± 0.134	0.134 ± 0.180	0.011*^,a,b^	0.591	0.280
Left Anterior Dorsal Putamen	0.419 ± 0.177	0.295 ± 0.165	0.261 ± 0.199	0.002**^,a,b^	0.802	0.229
Left Anterior Ventral Putamen	0.374 ± 0.209	0.302 ± 0.193	0.207 ± 0.217	0.007**^,a^	0.777	0.363
Left Posterior Dorsal Putamen	0.492 ± 0.215	0.380 ± 0.154	0.324 ± 0.241	0.006**^,a^	0.986	0.401
Left Posterior Ventral Putamen	0.507 ± 0.244	0.456 ± 0.210	0.373 ± 0.316	0.124	0.910	0.204
Left Thalamus	0.395 ± 0.244	0.227 ± 0.139	0.260 ± 0.233	0.011*^,a,b^	0.491	0.956
Right SMA	0.421 ± 0.278	0.396 ± 0.265	0.407 ± 0.305	0.823	0.099	0.334
Right Caudate	0.258 ± 0.221	0.132 ± 0.153	0.130 ± 0.187	0.013*^,a,b^	0.703	0.356
Right Anterior Dorsal Putamen	0.432 ± 0.214	0.313 ± 0.159	0.258 ± 0.201	0.001**^,a^	0.887	0.208
Right Anterior Ventral Putamen	0.353 ± 0.256	0.278 ± 0.201	0.191 ± 0.235	0.020*^,a^	0.755	0.716
Right Posterior Dorsal Putamen	0.466 ± 0.184	0.337 ± 0.141	0.320 ± 0.234	0.006**^,a,b^	0.871	0.617
Right Posterior Ventral Putamen	0.439 ± 0.267	0.350 ± 0.180	0.349 ± 0.310	0.313	0.780	0.248
Right Thalamus	0.359 ± 0.228	0.217 ± 0.142	0.244 ± 0.254	0.037*	0.296	0.925
Right Superior Cerebellum	0.675 ± 0.329	0.519 ± 0.295	0.443 ± 0.268	0.005**^,a,b^	0.858	0.696

BOLD activity means (SD) from each brain ROI. A repeated measures MANCOVA covarying for age, sex and handedness was performed to determine if there are group differences. The main effect of diagnosis, scan and the interaction between scan and diagnosis for each brain region. Significance indicated by *P < 0.05 and **P < 0.01. Pairwise *post hoc* analysis when *P*_FDR_ < 0.05. ^a^Controls versus Parkinson’s disease. ^b^Controls versus RBD. ^c^RBD versus Parkinson’s disease.

FDR, false discovery rate; MANCOVA, multivariate analysis of covariance; M1, primary motor cortex; RBD, rapid eye movement behaviour disorder; SMA, supplementary motor area; SD, standard deviation.

### Free-water imaging

**Figure 3:**
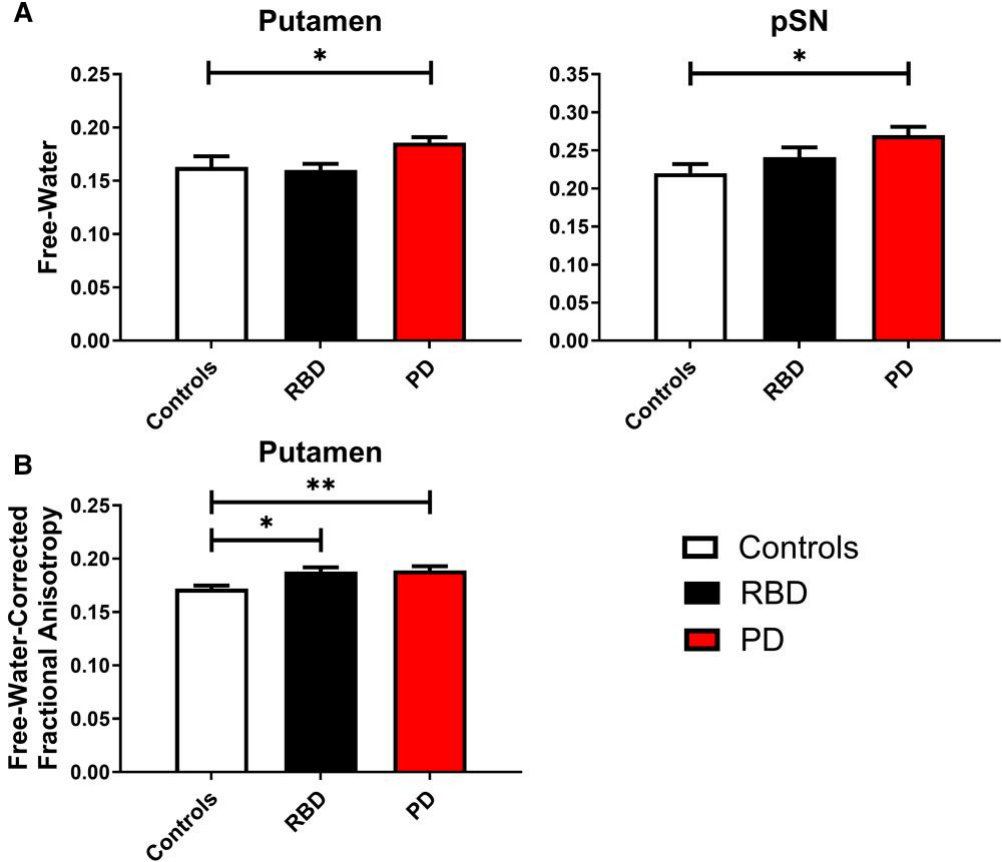
**Free-water imaging *post hoc*.** (**A**) Free-water group means (± 1 SEM) for the putamen and pSN. (**B**) Free-water-corrected fractional anisotropy group mean (± 1 SEM) for the putamen. Significance indicated by *P_FDR_ < 0.05 and **P_FDR_ < 0.01. Note: MANCOVA was used covarying for age and sex. Controls (*n* = 25). RBD (*n* = 24). Parkinson’s disease (*n* = 39). FDR, false discovery rate; MANCOVA, multivariate analysis of covariance; PD, Parkinson’s disease; pSN, posterior substantia nigra; RBD, rapid eye movement behaviour disorder; SEM, standard error of the mean.

To evaluate significant differences in free-water imaging (free-water and FA_T_), a MANCOVA was used covarying for age and sex. We found a significant difference between groups for the overall MANCOVA model [*F*(12,154) = 2.072, *P* = 0.022, Wilks’ Λ = 0.741] (Table 3). Figure 3A depicts group differences in free-water in the putamen and pSN (*P* < 0.05). In *post hoc* analysis, we found that the free-water putamen ROI (*P* < 0.05) showed that Parkinson’s disease had significantly greater free-water compared with controls (*P*_FDR_ = 0.025) and RBD (*P*_FDR_ = 0.025). The pSN free-water ROI (*P* < 0.05) revealed that the Parkinson’s disease group had significantly greater free-water compared with controls (*P*_FDR_ = 0.012). There were no group differences in free-water in the caudate (*P* > 0.05). Figure 3B depicts group differences in putamen FA_T_ (*P* < 0.05). In *post hoc* analysis, controls have significantly lower FA_T_ values compared with Parkinson’s disease (*P*_FDR_ = 0.008) and RBD (*P*_FDR_ = 0.020). There were no group differences in FA_T_ for the caudate or pSN (*P* > 0.05).

**Table 3:** Free-water imaging data

Brain region of interest	Controls (*n* = 25)	RBD (*n* = 24)	Parkinson’s disease (*n* = 38)	*F*-statistic	*P*-value
Free-water					
Caudate	0.278 ± 0.047	0.279 ± 0.041	0.297 ± 0.034	2.245	0.112
Putamen	0.163 ± 0.048	0.160 ± 0.031	0.186 ± 0.030	4.771	0.011*^,a,c^
pSN	0.220 ± 0.059	0.241 ± 0.063	0.270 ± 0.070	4.257	0.017*^,a^
Free-water-corrected fractional anisotropy					
Caudate	0.227 ± 0.026	0.227 ± 0.017	0.232 ± 0.021	0.924	0.401
Putamen	0.172 ± 0.017	0.188 ± 0.018	0.189 ± 0.027	4.670	0.012*^,a,b^
pSN	0.625 ± 0.029	0.629 ± 0.032	0.631 ± 0.037	0.116	0.890

Free-water and free-water-corrected fractional anisotropy means (SD) from each brain ROI. MANCOVA was performed covarying for age and sex to determine if there were group differences. The main effect of diagnosis of each brain region. Significance indicated by *P < 0.05. Pairwise *post hoc* analysis when *P* < 0.05 (FDR corrected). ^a^Controls versus Parkinson’s disease. ^b^Controls versus RBD. ^c^RBD versus Parkinson’s disease.

FDR, false discovery rate; MANCOVA, multivariate analysis of covariance; pSN, posterior substantia nigra; RBD, rapid eye movement behaviour disorder; SD, standard deviation.

### Purdue Pegboard Test

**Figure 4:**
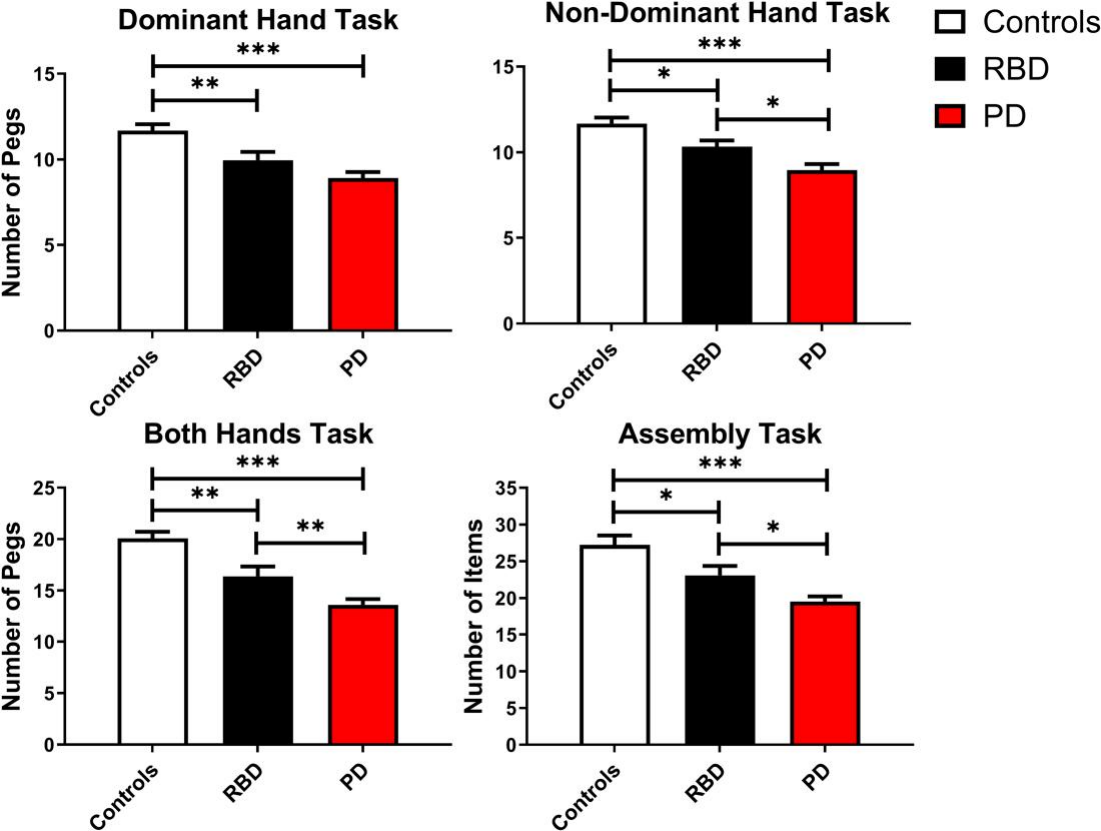
**PPT tasks.** Group mean (± 1 SEM) for the dominant hand task, non-dominant hand task, both hands task and assembly task. Significance indicated by *P_FDR_ < 0.05, **P_FDR_ < 0.01 and ***P_FDR_ < 0.001. Note: MANCOVA was used covarying for age and sex. Controls (*n* = 25). RBD (*n* = 24). Parkinson’s disease (*n* = 39). FDR, false discovery rate; MANCOVA, multivariate analysis of covariance; PD, Parkinson’s disease; RBD, rapid eye movement behaviour disorder; SEM, standard error of the mean.

To evaluate significant differences in PPT, a MANCOVA was used covarying for age and sex. We found that there was a significant difference between groups for the overall MANCOVA model [*F*(8,162) = 5.475, *P* < 0.001, Wilks’ Λ = 0.616]. We found that all PPT tasks were significantly different (*P*’s < 0.001) between groups (Table 4). In *post hoc* analysis (Fig. 4), participants with RBD performed significantly worse on the dominant hand and both hands tasks (*P*_FDR_’s < 0.01) and the non-dominant hand and assembly tasks (*P*_FDR_’s < 0.05) compared with controls. Participants with Parkinson’s disease performed significantly worse on all PPT tasks (*P*_FDR_’s < 0.001) compared with controls. Finally, participants with Parkinson’s disease performed significantly worse on the non-dominant hand task, both hands task and assembly task compared with RBD (*P*_FDR_’s < 0.05). We evaluated the effect size to determine which task of the PPT was the most sensitive in differentiating between groups. We found that the both hands task had an effect size $(\mathrm{partial}\;\eta_{p}^{2}=0.355)\;$ that was greater than the dominant hand task $\;(\mathrm{partial}\;\eta_{p}^{2}=0.223)$, non-dominant hand task $\;(\mathrm{partial}\;\eta_{p}^{2}=0.253)$ and assembly task $\;(\mathrm{partial}\;\eta_{p}^{2}=0.285)$.

**Table 4:** Purdue Pegboard Test

Purdue Pegboard task	Controls (*n* = 25)	RBD (*n* = 24)	Parkinson’s disease (*n* = 39)	*F*-statistic	*P*-value
Dominant hand	11.68 ± 1.887	9.94 ± 2.338	8.92 ± 2.157	11.93	<0.001***^,a,b^
Non-dominant hand	11.68 ± 1.749	10.33 ± 1.847	8.97 ± 2.194	14.08	<0.001***^,a,b,c^
Both hands	20.08 ± 3.226	16.89 ± 4.057	13.62 ± 3.529	22.84	<0.001***^,a,b,c^
Assembly	27.24 ± 6.405	23.22 ± 6.339	19.54 ± 4.248	16.52	<0.001***^,a,b,c^

Participant PPT mean scores (SD) for each task: dominant hand, non-dominant hand, both hands together and assembly. MANCOVA was run with age and sex as covariates to determine if there were differences between groups. The main effect of diagnosis is listed. Significance indicated by *** when *P* < 0.001. Pairwise *post hoc* analysis when *P* < 0.05 (FDR corrected): ^a^Controls versus Parkinson’s disease. ^b^Controls versus RBD. ^c^RBD versus Parkinson’s disease.

FDR, false discovery rate; MANCOVA, multivariate analysis of covariance; RBD, rapid eye movement behaviour disorder; SD, standard deviation.

### Relation between PPT score and BOLD imaging

We performed a backwards linear regression for each group to observe the relation between the both hands task of the PPT and PD-CON-BOLD ROIs. The regression for controls yielded a significant model with 1 significant factor, the right anterior ventral putamen (Supplementary Table 1). The regression for RBD yielded a significant model with 5 significant factors including the left M1, left anterior ventral putamen, right anterior dorsal putamen, right posterior dorsal putamen and age (Supplementary Table 1). The regression for Parkinson’s disease revealed a significant model with 4 significant factors, including the left anterior ventral putamen, left thalamus, right superior cerebellum and age (Supplementary Table 1).

### Relation between MDS-UPDRS-III score and BOLD imaging

We performed a separate backwards linear regression for each group to observe the relation between scores on the MDS-UPDRS-III and PD-CON-BOLD ROIs. The regression for controls yielded a significant model with 5 significant factors, the left anterior dorsal putamen, left anterior ventral putamen, left posterior dorsal putamen, left thalamus and age (Supplementary Table 2). The regression for RBD yielded a significant model with 2 significant factors, the left anterior dorsal putamen and age (Supplementary Table 2). The regression for Parkinson’s disease yielded a significant model with 6 significant factors, including left M1, left anterior dorsal putamen, left anterior ventral putamen, right anterior dorsal putamen, right anterior venteral putamen and right posterior dorsal putamen (Supplementary Table 2).

### Relation between PPT score and free-water imaging

We performed a backwards linear regression for each group using the significant PD-CON-FW-FA_T_ ROIs, as well as age and sex to observe the relation between the both hands task of the PPT and PD-CON-FW-FA_T_ ROIs. The Parkinson’s disease group did not yield a significant model [adjusted *R*^2^ = 0.033, *F*(1,36) = 2.247, *P* = 0.143]. The regression for controls yielded a significant model [adjusted *R*^2^ = 0.195, *F*(1,23) = 6.808, *P* = 0.016] with age as the only significant factor (*β* = −0.478, *P* = 0.016, VIF = 1.000). The regression for RBD yielded a significant model [adjusted *R*^2^ = 0.180, *F*(1,22) = 6.048, *P* = 0.022] with putamen FA_T_ as the only significant factor (*β* = −0.464, *P* = 0.022, VIF = 1.000).

### Relation between MDS-UPDRS-III score and free-water imaging

We performed a separate backwards linear regression for each group using the significant PD-CON-FW-FA_T_ ROIs, as well as age and sex to observe the relation between the MDS-UPDRS-III score and PD-CON-FW-FA_T_ ROIs. The regression for controls [adjusted *R*^2^ = −0.031, *F*(1,23) = 0.279, *P* = 0.602] and RBD [adjusted *R*^2^ = 0.026, *F*(1,22) = 1.618, *P* = 0.217] did not yield a significant model. The regression for Parkinson’s disease yielded a significant model with 1 significant factor [adjusted *R*^2^ = 0.127, *F*(2,35) = 3.679, *P* = 0.035], which was the pSN free-water (*β* = 0.460, *P* = 0.012, VIF = 1.278).

### Imaging and behavioural data early-stage RBD cohort

Demographic statistics of the RBD cohort (<2 years of diagnosis) are summarized in Supplementary Table 3. There was no significant group difference in age (*P* = 0.212), MVC (*P* = 0.629), sex (*P* = 0.057), handedness (*P* = 0.374) or MDS-UPDRS-III score (*P* = 0.629). We found that early-stage RBD has significantly higher RBDSQ score (*P* < 0.001) compared with controls. We also found that early-stage RBD had significantly lower MOCA scores (*P* = 0.030) compared with controls. All other pairwise *post hoc* comparisons did not significantly differ (*P*_FDR_ > 0.05).

To evaluate significant differences in the BOLD response, we performed a two-factor [group (2) × scan (3)] repeated measures MANCOVA covarying for age, sex and handedness. The main effect of scan [*F*(32,6) = 1.225, *P* = 0.434, Wilks’ Λ = 0.133] and the interaction between scan and diagnosis [*F*(32,6) = 2.214, *P* = 0.162, Wilks’ Λ = 0.078] were not significant. There was a statistically significant difference between groups for the overall MANCOVA model [*F*(16,22) = 2.893, *P* = 0.011, Wilks’ Λ = 0.322]. The following ROIs were significantly lower in early-stage RBD compared with controls left M1, left caudate, left anterior dorsal putamen, left thalamus right caudate, right anterior dorsal putamen, right posterior dorsal putamen, and right thalamus were significantly lower (*P*’s < 0.05) (Supplementary Table 3).

We evaluated differences in free-water imaging (free-water and FA_T_) using a MANCOVA covarying for age and sex. We did not find a significant difference between groups for the overall MANCOVA model [*F*(6,33) = 2.042, *P* = 0.088, Wilks’ Λ = 0.729].

To evaluate differences in PPT performance, a MANCOVA was used covarying for age and sex. We found that there was a statistically significant difference between groups for the overall MANCOVA model [*F*(4,35) = 2.881, *P* = 0.037, Wilks’ Λ = 0.752]. In *post hoc* analysis, we found that early-stage RBD had lower scores on all PPT tasks (*P*’s < 0.05) compared with controls (Supplementary Table 3).

### Parkinson’s disease most affected side cohort

Demographic statistics for controls, RBD, PD-Dom-Dom and PD-Dom-NonDom are summarized in Supplementary Table 4. There was no group difference in age (*P* = 0.619), MVC (*P* = 0.636), sex (*P* = 0.171) or handedness (*P* = 0.299). We found group differences in RBDSQ score, MOCA score and MDS-UPDRS-III score (*P*’s < 0.05). In *post hoc* analysis, we found that controls had significantly lower RBDSQ scores compared with RBD (*P*_FDR_ < 0.001), PD-Dom-Dom (*P*_FDR_ = 0.036) and PD-Dom-NonDom (*P*_FDR_ = 0.007). RBD had significantly higher RBDSQ scores compared with PD-Dom-Dom and PD-Dom-NonDom (*P*_FDR_’s < 0.001). PD-Dom-Dom and PD-Dom-NonDom had significantly higher MDS-UPDRS-III scores compared with controls and RBD (*P*_FDR_’s < 0.001). RBD had significantly higher MDS-UPDRS-III scores compared with controls (*P*_FDR_ = 0.037). All other pairwise *post hoc* comparisons were not significant (*P*_FDR_ > 0.05).

To evaluate significant differences on PPT score, a MANCOVA was used covarying for age and sex. There was a significant difference between groups for the overall MANCOVA model [*F*(12,209.3) = 4.181, *P* < 0.001, Wilks’ Λ = 4.181]. We found that all PPT tasks were significantly different (*P*’s < 0.001) between groups (Supplementary Table 4). In *post hoc* analysis, controls had a significantly higher PPT score on all tasks compared with RBD (*P*’s < 0.05), PD-Dom-Dom and PD-Dom-NonDom (*P*’s < 0.001). During the non-dominant hand task, PD-Dom-NonDom performed significantly worse compared with PD-Dom-Dom and RBD (*P*’s < 0.05). In the both hands task, RBD performed significantly better than PD-Dom-Dom (*P* = 0.011) and PD-Dom-NonDom (*P* = 0.001). RBD performed significantly better than PD-Dom-NonDom (*P* = 0.014) during the assembly task.

## Discussion

In this study, we explored the location of functional and free-water imaging changes in the brain of RBD participants, in comparison with controls and early-stage Parkinson’s disease. The strength of this study was that the RBD participants were polysomnography confirmed and did not have another neurological diagnosis, and 70.1% were within 2 years of an RBD diagnosis. We identified several key findings. (i) Both RBD and Parkinson’s disease groups had a significant reduction in BOLD signal in the left M1, left caudate, left putamen, left thalamus, right caudate and right putamen compared with controls. In the subset of RBD with <2 years of diagnosis, the same ROIs showed a reduction in BOLD signal compared with controls. (ii) There was an increase in FA_T_ in the putamen in both RBD and Parkinson’s disease compared with controls, with a similar observation for the RBD group with <2 years since diagnosis. (iii) There was a decline in PPT performance in all tasks in RBD and Parkinson’s disease compared with controls, and this observation held for the RBD group with <2 years since diagnosis. (iv) M1 and putamen BOLD activity were predictors for PPT score (both hands task) in the RBD cohort.

In Parkinson’s disease, prior work has shown reduced activity in the motor cortex, caudate, putamen, thalamus and cerebellum compared with controls using task-based BOLD fMRI.^16-24^ Oltra *et al*.^77^ found evidence that there is disrupted functional connectivity in Parkinson’s disease participants with probable RBD compared with controls. Prior work has also shown that RBD has reduced striatal DAT binding compared with controls and higher striatal DAT binding compared with Parkinson’s disease without an RBD diagnosis.^34,78,79^ There is also evidence of reduced striatal DAT binding longitudinally over 3 years in RBD compared with controls.^36^ Additionally, a prior study using ^11^C-MeNER and ^18^F-DOPA PET imaging found that individuals with RBD have a reduction of sensorimotor cortex ^11^C-MeNER binding compared with controls. The authors also found that within RBD participants, a significant correlation exists between thalamic ^11^C-MeNER binding and putaminal ^18^F-DOPA uptake.^80^ Stokholm *et al*.^81^ have also found that RBD participants have reduced ^18^F-DOPA Ki values in the thalamus compared with controls. The novel contribution of the current study is that RBD participants have reduced BOLD signal in the motor cortex, caudate, putamen and thalamus compared with controls. These observations were also found when focused on RBD participants within 2 years of diagnosis. This set of findings provides new insight into how neuronal function is compromised in RBD and that these observations seem to be occurring in the early stages of RBD post-diagnosis.

A robust finding in Parkinson’s disease is that there is elevated free-water in the substantia nigra and that free-water increases longitudinally over 1, 2 and 4 years in early Parkinson’s disease.^42,43,47,82,83^ The current study found support for this literature, in that Parkinson’s disease has elevated free-water in the pSN compared with controls. Prior studies have reported that RBD participants can have elevated free-water in the pSN,^44-46^ although no significant difference was found in the current study. One plausible explanation for this difference is due to the mean age of the RBD participants. The current study included individuals with RBD with a mean age of 60.3 years old while previous studies included individuals with RBD with a mean age between 63 and 66 years old.^44-46^ In our study, the RBD group did have a 9% increase in free-water compared with the control group, but the change was not significant. It is possible that longitudinally the free-water could increase for the RBD group studied here, and more work is needed to address this interesting question.

A novel set of findings in this study were the free-water imaging changes in the putamen for both RBD and Parkinson’s disease groups, compared with the control group. While free-water was elevated within the putamen for the Parkinson’s disease cohort, only FA_T_ was increased in both the RBD and Parkinson’s disease groups. It is possible that tissue degeneration could allow for more free-water to diffuse within interstitial spaces, where reactive astrocytes assemble around the injury site and increase tissue anisotropy.^48,84^ Since the RBD cohort and Parkinson’s disease cohort have elevated FA_T_ in the putamen, and only the Parkinson’s disease group had elevated free-water in the putamen, it is possible to hypothesize that increased FA_T_ is occurring prior to elevated free-water. If elevated FA_T_ is an acute response to an injury as shown in prior work,^48^ it is possible that an acute event could occur and then normalize once free-water is elevated. In a prior study of individuals with PSP, it was observed that pSN and caudate nucleus have elevated free-water whereas FA_T_ was not elevated, consistent with the hypothesis that increased free-water can occur without increased FA_T_.^85^

Previous work has demonstrated that the PPT is an inexpensive and reliable method to classify upper extremity motor deficits in individuals with Parkinson’s disease and RBD.^51-57^ In some studies, the authors have summed individual scores of the PPT tasks, although there is not a general consensus on which composite score to use.^55-57^ In the current study, we observed that individuals with RBD and Parkinson’s disease had a significant decline in PPT performance in all four tasks. Additionally, the both hands task was the most sensitive task to distinguish controls from RBD and controls from Parkinson’s disease. This observation is consistent with previous findings that the both hands task was the most sensitive to distinguish between controls, Parkinson’s disease and atypical Parkinsonism’s (Multiple System Atrophy and Progressive Supranuscular Palsy).^51^

In the literature, there has been an association between neuroimaging measures and MDS-UPDRS and the both hands task score of the PPT. Prior work in Parkinson’s disease has shown within the striatum the DAT-specific binding ratio was associated with MDS-UPDRS score.^41,86,87^ In this study, we found that for the RBD group, the putamen BOLD ROI was able to predict MDS-UPDRS-III score. In addition, the putamen and motor cortex BOLD ROIs and pSN free-water ROI were able to predict MDS-UPDRS-III score for the Parkinson’s disease groups. Wilkes *et al*.^51^ found that in Parkinson’s disease, the structural free-water ROIs (pSN free-water, putamen free-water, pedunculopontine FA_T_, pSN FA_T_ and subthalamic nucleus FA_T_) were able to predict PPT both hands score. A novel contribution to this study is that BOLD imaging ROIs (motor cortex and putamen) and free-water imaging (FA_T_ of the putamen) were able to predict the PPT both hands scores for the RBD group. BOLD imaging ROIs (putamen, thalamus and cerebellum) were also able to predict PPT both hands score for the Parkinson’s disease group.

There is not a general consensus on how to define early-stage RBD. In the current study, we explored a cohort of RBD participants within 2 years of diagnosis. However, RBD could exist prior to diagnosis, and the time to diagnosis could be almost a decade for some patients.^88^ Based on the literature, a bed partner is a crucial factor in why an individual seeks medical advice.^88-90^ The conversion rate from RBD to an overt neurodegenerative syndrome was 73.5% after a 12-year follow-up.^10^ Thus, it could be almost two decades between symptom onset and transition to a neurodegenerative disease diagnosis. In the review by Mitchell *et al*.,^31^ it was noted that there are few longitudinal studies from the early stages of RBD that may provide key insights into the physiological and neurodegenerative changes that occur. The current study provides new findings that fMRI BOLD and free-water imaging changes exist within the basal ganglia within 2 years of an RBD diagnosis.

## Conclusion

In conclusion, the current study revealed distinct patterns of functional, microstructure and behavioural changes in RBD and Parkinson’s disease. Specifically, there was decreased functional activity in both RBD and early-stage Parkinson’s disease within the motor cortex, putamen, caudate and thalamus, providing new evidence that these areas may be the first neuroanatomical regions to undergo functional changes in a synucleinopathy. We also found microstructural changes demonstrated by elevated FA_T_ in the putamen in RBD and Parkinson’s disease and elevated free-water in the putamen and pSN in Parkinson’s disease compared with controls. This suggests the putamen and the pSN may be the first neuroanatomical regions to undergo microstructural changes in RBD and Parkinson’s disease. We also observed the both hands task of the PPT was most sensitive in distinguishing between controls, RBD and Parkinson’s disease. It will be up to future work to study RBD longitudinally to assess changes in both functional and free-water imaging to examine the stability of these markers over time.

## Supplementary Material

fcae344_Supplementary_Data

## Data Availability

The deidentified data and analysis code may be shared upon reasonable request. The code used was part of publicly available software, including Analysis of Functional Neuroimages, FSL, R and R Studio.
