# Supplementary material for: Functional and free-water imaging in rapid eye movement behaviour disorder and Parkinson’s disease
Source: Brain Commun. 2024 Oct 10;6(5):fcae344. doi: 10.1093/braincomms/fcae344 (PMC11474242; doi:10.1093/braincomms/fcae344)
Supplement: fcae344_Supplementary_Data [file fcae344_supplementary_data.docx]

**Supplementary Table 1. Relation Between Purdue Pegboard Test Score and Functional Imaging.** Backwards linear regression was used to determine if there is a relationship between on the PPT both hands task and functional imaging in healthy controls and individuals with RBD and Parkinson’s Disease. Significance indicated by * p<0.05, ** p<0.01, and *** p<0.001.

|  | **Standardized**  **Beta Coefficient** | **T-Statistic** | **p-value** | **Collinearity Statistics (VIF)** |
| --- | --- | --- | --- | --- |
| **Controls**  **[Adjusted R^2^=0.375, F(5,19)=3.875, p=0.014]** | |  |  |  |
| *Imaging* |  |  |  |  |
| Right Anterior Ventral Putamen | 0.909 | 2.507 | 0.021* | 5.046 |
| **RBD**  **[Adjusted R^2^=0.595, F(6,17)=6.635, p<0.001]** | |  |  |  |
| *Imaging* | |  |  |  |
| Left M1 | 0.517 | 3.090 | 0.007** | 1.591 |
| Left Anterior Ventral Putamen | -0.970 | -3.458 | 0.003** | 4.471 |
| Right Anterior Ventral Putamen | 0.830 | 3.052 | 0.007** | 4.197 |
| Right Posterior Dorsal Putamen | 0.378 | 2.220 | 0.040* | 1.645 |
| *Demographic* |  |  |  |  |
| Age | -0.406 | -2.590 | 0.019* | 1.397 |
| **Parkinson’s Disease**  **[Adjusted R^2^=0.496, F(7,31)=6336, p<0.001]** | |  |  |  |
| *Imaging* | |  |  |  |
| Left Anterior Ventral Putamen | 0.764 | 2.817 | 0.008** | 5.548 |
| Left Thalamus | -0.492 | -2.673 | 0.012* | 2.556 |
| Right Cerebellum | 0.988 | 6.076 | <0.001*** | 1.992 |
| *Demographic* |  |  |  |  |
| Age | -0.326 | -2.618 | 0.014* | 1.166 |

Abbreviations: BOLD = blood-oxygen-level-dependent; M1= primary motor cortex; RBD = Rapid Eye Movement Behavior Disorder

**Supplementary Table 2. Relation between MDS-UPDRS-III Score and Functional Imaging.** Backwards linear regression was used to determine if there is a relationship between MDS-UPDRS-III score and functional imaging in healthy controls and individuals with RBD and Parkinson’s Disease. Significance indicated by * p<0.05, ** p<0.01, and *** p<0.001.

|  | **Standardized**  **Beta Coefficient** | **T-Statistic** | **p-value** | **Collinearity Statistics (VIF)** |
| --- | --- | --- | --- | --- |
| **Controls**  **[Adjusted R^2^= 0.727, F(9,15)= 8.117, p<0.001]** | |  |  |  |
| *Imaging* |  |  |  |  |
| Left Anterior Dorsal Putamen | -1.015 | -3.671 | 0.002** | 6.733 |
| Left Anterior Ventral Putamen | -0.672 | -3.123 | 0.007** | 4.080 |
| Left Posterior Dorsal Putamen | 0.597 | 2.667 | 0.018* | 4.410 |
| Left Thalamus | 0.936 | 3.635 | 0.002** | 5.838 |
| *Demographic* |  |  |  |  |
| Age | -0.725 | -3.972 | 0.001** | 2.930 |
| **RBD**  **[Adjusted R^2^ = 0.317, F(4,19)= 3.672, p=0.022]** | |  |  |  |
| *Imaging* |  |  |  |  |
| Left Anterior Dorsal Putamen | -0.799 | -2.534 | 0.020* | 3.354 |
| *Demographic* |  |  |  |  |
| Age | 0.473 | 2.413 | 0.026* | 1.297 |
| **Parkinson’s Disease**  **[Adjusted R^2^ = 0.595, F(6,32) = 10.30, p<0.001]** | |  |  |  |
| *Imaging* |  |  |  |  |
| Left M1 | 0.797 | 5.467 | 0.001*** | 1.991 |
| Left Anterior Dorsal Putamen | 0.597 | 2.618 | 0.013* | 4.876 |
| Left Anterior Ventral Putamen | -1.064 | -3.951 | <0.001*** | 6.806 |
| Right Anterior Dorsal Putamen | -1.723 | -6.225 | 0.001*** | 7.189 |
| Right Anterior Ventral Putamen | 0.967 | 3.168 | 0.003** | 8.739 |
| Right Posterior Dorsal Putamen | 0.581 | 2.747 | 0.010* | 4.204 |

Abbreviations: BOLD = blood-oxygen-level-dependent; M1= primary motor cortex; RBD = Rapid Eye Movement Behavior Disorder

**Supplementary Table 3. Early-Stage RBD vs Controls.** Demographics of early-stage RBD cohort compared to controls. Significant functional imaging regions of interest (mean ± SD) between early-stage RBD and controls. Purdue Pegboard Test (mean ± SD) significant tasks between early-stage RBD and controls. One-way ANOVA was performed for continuous demographic data and Fisher’s Exact test to determine if there were differences between groups. Repeated measures MANCOVA covarying for age, sex, and handedness was used for functional imaging to determine if there were differences between groups. MANCOVA covarying for age and sex was used for the Purdue Pegboard Test. Significance indicated by *p<0.05, **<0.01, and ***p<0.001.

|  | **Controls**  (n=25) | **Early-Stage RBD**  (n=17) | **F-statistic** | **p-value** |
| --- | --- | --- | --- | --- |
| **Demographics** |  |  |  |  |
| Age | 62.56 ± 7.880 | 59.29 ± 8.630 | - | 0.212 |
| MVC | 79.90 ± 23.01 | 83.94 ± 29.32 | - | 0.629 |
| Sex | 11M / 14F | 13M /4F | - | 0.057 |
| Handedness | 5L / 20R | 0L / 27R |  | 0.374 |
| RBDSQ | 1.400 ± 1.607 | 7.350 ± 2.396 | - | <0.001*** |
| MOCA | 27.00 ± 2.179 | 25.21 ± 3.238 | - | 0.030* |
| MDS-UPDRS-III | 4.320 ± 3.301 | 6.290 ± 4.793 | - | 0.629 |
| **Functional Imaging**  **[F(16,22)=2.893, p=0.011, Wilks’ Ʌ=0.322]** | | |  |  |
| Left M1 | 0.693 ± 0.372 | 0.427 ± 0.294 | 9.059 | 0.005** |
| Left Caudate | 0.255 ± 0.205 | 0.144 ± 0.154 | 6.595 | 0.014* |
| Left Anterior Dorsal Putamen | 0.419 ± 0.178 | 0.283 ± 0.147 | 8.738 | 0.005* |
| Left Thalamus | 0.395 ± 0.245 | 0.225 ± 0.156 | 7.307 | 0.010* |
| Right Caudate | 0.258 ± 0.221 | 0.133 ± 0.175 | 6.551 | 0.015* |
| Right Anterior Dorsal Putamen | 0.432 ± 0.214 | 0.298 ± 0.15 | 6.835 | 0.013* |
| Right Posterior Dorsal Putamen | 0.466 ± 0.184 | 0.319 ± 0.113 | 10.551 | 0.002** |
| Right Thalamus | 0.359 ± 0.228 | 0.224 ± 0.162 | 5.175 | 0.029* |
| **Purdue Pegboard Test Task**  **[F(4,35)=2.881, p=0.037, Wilks’ Ʌ=0.752]** | | |  |  |
| Dominant Hand | 11.68 ± 1.887 | 9.76 ± 1.954 | 12.037 | 0.001** |
| Non-Dominant Hand | 11.68 ± 1.749 | 10.47 ± 1.772 | 6.176 | 0.017* |
| Both Hands | 19.8 ± 3.122 | 17.18 ± 3.957 | 8.557 | 0.006** |
| Assembly | 27.48 ± 6.312 | 23.82 ± 5.703 | 5.302 | 0.027* |

Abbreviations: F = female; FDR= false-discovery rate; MDS-UPDRS-III = Movement Disorders Society – Unified Parkinson’s Disease Rating Scale – Part 3; M = male; M1= primary motor cortex; MOCA = Montreal Cognitive Assessment; MVC = maximum voluntary contraction; RBD = Rapid Eye Movement Behavior Disorder; RBDSQ = REM Sleep Behavior Disorder Questionnaire; SD = standard deviation

**Supplementary Table 4. Parkinson’s Disease Most Affected Side.** Demographics of controls, RBD, Parkinson’s Disease dominant hand side matches effected side, and Parkinson’s Disease dominant hand side and effected side do not match. Purdue Pegboard Test mean scores (SD) for each task: dominant hand, non-dominant hand, both hands together, and assembly for controls, RBD, Parkinson’s disease dominant hand side matches effected side, and Parkinson’s disease dominant hand side and effected side do not match. One-way ANOVA was performed for continuous demographic data and Fisher’s Exact test to determine if there were differences between groups. MANCOVA was performed for the PPT covarying for age and sex to determine if there are differences between groups. The main effect of diagnosis is listed. Significance indicated by *** when p<0.001.

|  | **Controls**  (n=25) | **RBD**  (n=24) | **PD-Dom-Dom**  (n=21) | **PD-Dom-NonDom**  (n=18) | **F-statistic** | **p-value** |
| --- | --- | --- | --- | --- | --- | --- |
| **Demographics** |  |  |  |  |  |  |
| Age | 62.56 ± 7.880 | 60.29 ± 8.595 | 62.62 ± 8.369 | 63.33 ± 7.252 | - | 0.619 |
| MVC | 79.90 ± 23.01 | 84.26 ± 28.14 | 85.81 ± 30.79 | 75.55 ± 27.21 | - | 0.636 |
| Sex | 11M / 14F | 18M /6F | 13M / 8F | 10M / 8F | - | 0.171 |
| Handedness | 20R / 5L | 24R / 0L | 17R / 4L | 17R / 1L | - | 0.240 |
| Disease Duration |  | 21.36 ± 27.83 | 28.17 ± 19.27 | 31.33 ± 15.19 | - | 0.299 |
| RBDSQ | 1.400 ± 1.607 | 7.458 ± 2.502 | 2.810 ± 1.965 | 3.333 ± 2.521 | - | <0.001*** |
| MOCA | 27.00± 2.179 | 25.25 ± 3.04 | 25.00 ± 2.665 | 25.78 ± 2.074 | - | 0.038* |
| MDS-UPDRS-III | 4.320 ± 3.301 | 8.208 ± 7.779 | 14.91 ± 5.735 | 18.61 ± 7.301 | - | <0.001*** |
| **Purdue Pegboard Test Tasks**  **[F(12, 209.3)= 4.181, p<0.001, Wilks’ Ʌ=4.181]** | | | |  |  |  |
| Dominant Hand | 11.68 ± 1.887 | 9.958 ± 2.368 | 8.762 ± 2.508 | 9.111 ± 1.711 | 12.04 | <0.001*** ^a,b,c^ |
| Non-Dominant Hand | 11.68 ± 1.749 | 10.33 ± 1.761 | 9.571 ± 1.859 | 8.278 ± 2.396 | 6.176 | <0.001*** ^a,b,c,d,f^ |
| Both Hands | 19.80 ± 3.122 | 17.08 ± 4.211 | 14.10 ± 3.285 | 13.06 ± 3.811 | 8.557 | <0.001*** ^a,b,c,e,f^ |
| Assembly | 27.48 ± 6.312 | 23.46 ± 6.171 | 20.19 ± 3.444 | 18.78 ± 5.024 | 5.302 | <0.001*** ^a,b,c,f^ |

Abbreviations: FDR= false-discovery rate; PD-Dom-Dom = Parkinson’s Disease Dominant Hand Side and Side Effected Matches; PD-Dom-NonDom = Parkinson’s Disease Dominant Hand Side and Effected Side Do Not Match; PPT = Purdue Pegboard Test; RBD = Rapid Eye Movement Behavior Disorder; SD standard deviation

Pairwise post-hoc analysis when p<0.05 (FDR corrected):

a = Controls versus PD Dom_Dom

b = Controls versus PD Dom_NonDom

c = Control versus RBD

d = PD Dom_Dom versus PD Dom_NonDom

e = PD Dom_Dom versus RBD

f = PD Dom_NonDom versus RBD
